# Are Adolescent Religious Attendance/Spirituality Associated with Family Characteristics?

**DOI:** 10.3390/ijerph16162947

**Published:** 2019-08-16

**Authors:** Klara Malinakova, Radek Trnka, Ludmila Bartuskova, Petr Glogar, Natalia Kascakova, Michal Kalman, Jitse P. van Dijk, Peter Tavel

**Affiliations:** 1 Olomouc University Social Health Institute, Palacký University Olomouc, 771 11 Olomouc, Czech Republic; 2 Prague College of Psychosocial Studies, Hekrova 805, 149 00 Prague 11, Czech Republic; 3 Faculty of Physical Culture, Institute of Active Living, Palacký University Olomouc, 771 11 Olomouc, Czech Republic; 4 Department of Community and Occupational Medicine, University Medical Center Groningen, University of Groningen, 9713 AV Groningen, The Netherlands; 5 Graduate School Kosice Institute for Society and Health, P.J. Safarik University in Kosice, 040 11 Kosice, Slovak Republic

**Keywords:** adolescent, family, communication, emotional support, parental monitoring, religion, spirituality

## Abstract

The family environment is associated with religiosity and spirituality as well as many aspects of adolescent lives, including their health behaviour. Therefore, the aim of this study was to assess family environment associations with adolescent religious attendance (RA), i.e., weekly participation in religious services, and spirituality in a highly secular country. A nationally representative sample (*n =* 4182, 14.4 ± 1.1 years, 48.6% boys) of Czech adolescents participated in the 2014 Health Behaviour in School-aged Children cross-sectional study. RA, spirituality and the family environment, i.e., family communication, perceived emotional support, and parental monitoring, were measured. Higher adolescent RA was associated with lower self-reported easiness of communication with mother (odds ratio (OR) = 0.68; 99% confidence interval (99% CI) = 0.47–0.99; *p* < 0.01). In contrast, spiritual respondents were more likely to report both easier communication with their father (OR per standard deviation (SD) change = 1.12, 99% CI 1.02–1.23; *p* < 0.01) and mother (OR per SD change = 1.38 (1.23–1.55); *p* < 0.001) and higher perceived emotional support (OR per SD change = 1.73 (1.55–1.92); *p* < 0.001). Parents of respondents who attended religious services at least once a week, as well as parents of spiritual respondents, were generally more likely to monitor adolescent behaviour. Thus, this study provides information for parents, mental health workers, and pastoral carers. Further research should assess the association of a lower easiness of family communication with dissonances in adolescent–parent religiosity/spirituality and with higher parental monitoring.

## 1. Introduction

Religion and spirituality (R/S) are generally suggested to be associated with the higher well-being of individuals [1]. Religiosity has been found to be a protective factor against risk behaviours such as use of drugs or alcohol in adolescence [2,3]. Furthermore, religious adolescents are less likely to partake in delinquent behaviours [4] and more likely to delay the onset of sexual activity [5]. Given all these positive outcomes, research of religiosity and spirituality within the primary family environment is considered to be exceptionally important. During the sensitive period of adolescence, religiosity and spirituality in the family may have some positive impacts on adolescent health behaviour. However, it is not clear what role the family environment and family communication play in relation to the development of religiosity and spirituality in adolescents.

Before proceeding to the overview of family communication patterns and parenting styles, the difference between religiosity and spirituality should first be briefly outlined. Religiosity is defined in terms of behaviour, such as church attendance [6], whereas spirituality is understood to be an internal individual contentedness, one’s perceived closeness to a Higher Power (e.g., God), a sense of the meaning of life, and of spiritual well-being [7]. Spirituality may also include nonreligious spiritual orientations, i.e., personal beliefs that are not specifically related to organized religion or religious teachings [6]. Zinnbauer and Pargament [8] summarized the definitions of religiosity and spirituality and concluded that the usefulness of polarizing religiosity and spirituality is unclear and that differences between the two will continue to be identified. In empirical research, religiosity and spirituality are sometimes treated as one factor in order to derive a composite score for general religiosity (e.g., [9]) and are sometimes compared with each other (e.g., [10]). Within the context of health-related outcomes of religiosity and spirituality in adolescents, the study of Malinakova et al. [10] showed that mere religious attendance and spirituality were associated with a decreased risk of only one or two kinds of health-risk behaviour. In contrast, their multiplicative interaction was associated with a decreased risk of four of the five health-risk behaviours. In other words, high spirituality protect adolescents from health-risk behaviour more if combined with religious practice.

As mentioned above, religiosity and spirituality in the family environment may have positive impact on adolescent health behaviour. For a better understanding of how religious attitudes and behaviours are shared and communicated within a family, the Family Communication Patterns Theory (FCPT) [11] provides us with a suitable framework for an operationalization of parent–child communication. According to the FCPT [11], families can be divided into four types based on their approach to family communication and family (social) conformity: (1) Consensual families with an emphasis on communication and conformity, (2) pluralistic families with an emphasis on communication, but not on conformity, (3) protective families with little emphasis on communication and big emphasis on conformity and respect towards authorities, and (4) laissez-faire families with little emphasis both on communication and conformity.

Despite the lack of research in the area, the communication of spiritual and religious attitudes may be expected to be influenced by the family atmosphere, conformity, or pluralism within family environment. Furthermore, parenting styles may also have a crucial impact on communication and the sharing of religious attitudes and behaviours within the family. Following Maccoby and Martin’s initial typology [12], different parenting styles can be distinguished, i.e., indulgent, authoritative, authoritarian, and neglectful. This typology has been widely used by past [13,14] as well as by more recent research [15,16].

Parental communication and the sharing of attitudes, including spiritual and religious ones, cannot be separated from family emotional support and monitoring. Parenting is closely connected with emotional support and control [12], and parents’ emotional characteristics, such as warmth, acceptance, attention, responsiveness, involvement, and support [17], are very important for the effective sharing of health-related attitudes and behaviours within the parent–adolescent relationship. Parental control and monitoring may have different qualities and may also be less or more adequate. Adequate parental control includes an adequate level of boundaries, demandingness, protection, and supervision [12], whereas less adequate parental control may include excessively coercive control, intrusion, and rejection.

The present study is focused on adolescence, which is a very sensitive period otherwise highly vulnerable for engaging in various risky behaviours. A lack of adolescent adjustment was found to be related with poor socialization outcomes in adulthood [18]. Adolescence is also a period during which peers are especially influential. Peer groups may have a negative impact on deviance [19] and even engagement in risky behaviours, such as suicide attempts or self-injury [20]. All these reasons make the investigation of parent–adolescent communication and the supportive role of family during adolescence especially important.

Several previous studies have explored the links between family communication and parents’ spirituality and religiosity. The study of Brelsford [21] investigated the use of spiritual disclosure and theistic and nontheistic sanctification of the parent in parent–adolescent dyads. Greater nontheistic sanctification and higher levels of spiritual disclosure were significantly related to increased parent–child relationship quality. In the study of Brelsford and Mahoney [22], the investigation of mother–adolescent dyads revealed that a greater spiritual disclosure was related to higher relationship satisfaction, greater use of collaborative conflict resolution strategies, less dysfunctional communication, less verbal aggression, and increased general disclosure. Hardy et al. [23] explored the socialization of religiousness and spirituality through the parenting styles used by the parents when the adolescents were younger. Family religiousness positively predicted individual religiousness and spirituality in later life, especially in families characterized by authoritative parenting [23]. In contrast, frequent, honest, and open communication with parents was more strongly and more significantly associated with adolescent spirituality than any specific parenting style [24]. Parents’ religiosity was also shown to be associated with lower adolescent risk behaviour via higher parental monitoring and higher adolescent self-control and religiosity [25].

Interestingly, the Czech Republic ranks among those European countries with a high percentage of adolescents perceiving difficulties in communication with parents. In their comparison of 12 European countries, Tabak et al. [26] state that in their 2005/2006 survey, 19.6% of Czech adolescents reported difficulty communicating with their mother and 38.7% difficulty communicating with their father. Higher percentages were reported only for France (22.3% for communication with the mother and 42.8% with father) and Switzerland (35.5% for communication with the father). The situation in the Czech Republic might also be conditioned by historical events and cultural determinants. The same might also be the case for the high percentage of religiously unaffiliated respondents, as the Czech Republic belongs among the most secular countries in the world [27]. This combination makes the Czech Republic an interesting research area. As the majority of research on R/S and the family environment was performed in mostly religious countries, research in a secular country can bring an interesting comparison and can raise questions regarding the generalizability of the previous findings. A better understanding of the associations of R/S with the adolescent family environment can help professionals in the area of adolescent mental health as well as pastoral carers.

To the best of our knowledge, there are only a few studies on this topic in the Czech Republic or in other highly secular countries. Therefore, the aim of this study was to assess the association between some family characteristics (family communication, perceived emotional support, and parental monitoring style) and adolescent religious attendance and spirituality in a highly secular country.

## 2. Materials and Methods

### 2.1. Participants and Procedure

We obtained data on a nationally representative sample of Czech boys and girls from the 2014 Health Behaviour in School-aged Children (HBSC) study. This cross-sectional World Health Organization collaborative study focused on health and health-related behaviour and their socioeconomic determinants in 11-, 13- and 15-year-old children. More detailed information about the survey can be found in Roberts et al. [28]. The HBSC-study has been conducted at 4-year intervals since 1983–1984 and now includes 44 countries across North America and Europe, including the Czech Republic. According to the HBSC study protocol, schools were selected randomly after stratification by region, school size, and type of school (primary schools vs. secondary schools). Out of 243 contacted schools, 242 schools agreed to participate (response rate 99.6%). Then, classes from the 5th, 7th, and 9th grades, in general corresponding to the age categories of 11-, 13- and 15-year-olds, were selected at random, one from each grade per school.

Data from 14,539 pupils were obtained (response rate 89.2%). Most non-response was due to illness or other reasons, e.g., sports or academic competitions (10.6%), and 30 children refused to participate in the survey (0.002%). The HBSC survey consists of mandatory items, which are obligatory for each country, optional items which can be chosen by each country from a common package, and finally a limited number of national items that can be specifically added by each country. Our R/S items belonged to this last category; therefore, due to a limited space, two versions of a survey were created. R/S was included only in version B of the survey, and only adolescents from the 7th and the 9th grades responded to these questions; so, for the purpose of this paper the dataset comprised 4889 adolescents. Because of incomplete information on age, gender, spirituality, or religiosity, or an age distinctly differing from the rest (we decided to include only the participants aged between 12.5 and 16.4, because this age cut-off corresponds to the age range that occurs in 7th and the 9th grade classes under normal conditions), 707 questionnaires were excluded, leading to a final sample of 4182 respondents (mean age = 14.43, SD = 1.07, 48.6% boys). Data was collected between April and June 2014. The questionnaires were distributed by trained administrators with no teachers present in the classroom in order to reduce information bias. Respondents had one class period (45 min) dedicated to completing the questionnaire. Participation in the survey was anonymous and voluntary.

The study design was approved by the Ethics Committee of the Faculty of Physical Culture, Palacký University Olomouc (No. 17/2013).

### 2.2. Measures

Religious attendance was assessed as an independent variable and was measured by the question “how often do you go to church or to religious sessions?” with possible answers of “several times a week, approximately once a week, approximately once a month, a few times a year, exceptionally, never”. This question was added into the survey as a national item. Sunday attendance is a matter of obligation in most of the churches/denominations in the Czech Republic; therefore, the participants who reported attending religious sessions at least once a week were dichotomized as attending.

Spirituality was measured using the adjusted shortened version of the Spiritual Well-Being Scale (SWBS) [29]. This scale was added into the survey as a national item. The scale measures the overall spiritual well-being and includes two subscales assessing religious and existential well-being. In the adjusted version [30], the Religious Well-Being Subscale (RWB) consists of four items that provide a self-assessment of one’s relationship with God (e.g., “I believe that God loves me and cares about me.”) while the other three form the Existential Well-Being Subscale (EWB), which gives a self-assessment measure of one’s sense of life purpose and life satisfaction (e.g., “I believe there is some real purpose for my life.”). Response possibilities for each item consisted of a 6-point Likert scale ranging from 1 (strongly disagree) to 6 (strongly agree). The overall score from the adjusted shortened SWBS is computed by summing the responses to all 7 items and ranges from 7 to 42, with a higher score representing greater spiritual well-being. In the main analysis, spirituality was assessed as a scale variable in order to prevent a loss of information. However, according to previous studies [10,31], spirituality was also dichotomized for sensitivity analysis (graphical representation), and participants with a score of 34 or higher (upper quartile of the score) were considered as spiritual, and the rest as non-spiritual. Cronbach’s alpha was 0.81 in our sample for the total scale, for the RWB it was 0.93, and for the EWB 0.76.

Family environment was assessed using the following variables: Communication with parents and perceived emotional help from the family. All of these items were assessed as independent variables and represented mandatory items in the survey.

Communication with parents was measured with the question: “How easy it is for you to talk to the persons listed below about things that really bother you?”, with the father and mother as separate options. Response options ranged from 1 (very easy) to 4 (very difficult), with a fifth option “I do not have or see this person”. According to the latest HBSC report [32], communication was dichotomized as “Easy” for those who answered “Very easy” or “Easy” and as “Difficult” for the remaining two answers.

Perceived family support was measured using the Multidimensional Scale of Perceived Social Support (MSPSS) family subscale [33], which is assessed with four items, e.g., “My family really tries to help me”. Response options ranged from 1 (very strongly disagree) to 7 (very strongly agree). In a binary logistic regression analysis, MSPSS was used as a dichotomized variable. According to the latest HBSC report [32], participants with a mean MSPSS score higher than 5.5 were considered to have a high family support, while the others as not having this. Cronbach’s alpha was 0.92 in our sample.

Parental monitoring was assessed by using nine statements on different kinds of family rules, where participants reported how often their parents control specific behaviour (parental monitoring of time on the TV, PC, and Internet; time spent out after school; regular breakfast) or allow specific behaviours (eating in front of the screen; sweets and soft drinks consumption; smoking and alcohol use) by using the response categories ranging from 1 (always) to 4 (never). All of these items were assessed as independent variables and represented mandatory items in the survey. For each item, parents reported by participants as being either always or usually controlling certain behaviour, or on the contrary, never or seldom allowing it, were dichotomized as monitoring.

Age, gender and socioeconomic status were considered as potential confounding variables. The socioeconomic status of the respondents’ families was used as a covariate and was assessed using the Family Affluence Scale (FAS) [34]. The scale examines the number of cars owned by the family, having one’s own bedroom, the number of computers in the household, the number of foreign family holidays, the number of bathrooms and dishwasher ownership. The summary score ranges from 10 to 13, and following HBSC recommendations [32], it was converted into a fractional rank (ridit) score, leading to transformation of ordinal data to an interval scale with a normalized range (from 0 to 1, with higher score indicating higher socioeconomic position) and distribution.

### 2.3. Statistical Analyses

First, the exploratory data analysis was performed, and the differences in basic characteristics and in the observed categorical variables were assessed using the Chi-Square test, comparing the groups of attending versus non-attending as well as spiritual versus non-spiritual respondents. Then, differences in spirituality levels between attending and non-attending respondents were compared using the Mann–Whitney U test. Consequently, the mutual relationship between all variables of this study was assessed with the Pearson correlation, using binary variables, or where available, scale variables.

In the next step, the associations between religious attendance, assessed as a dichotomized variable (Model 1), and spirituality, assessed as a continuous variable standardized to z-scores (Model 2), and family communication, perceived emotional support and nine types of family rules behaviour (parental monitoring of time on the TV, PC and Internet; time spent out after school; regular breakfast consumption; eating in front of a screen; sweets and soft drinks consumption; smoking and alcohol use) were analysed using a binary logistic regression models. The logistic regression was chosen because of the categorical nature of the dependent variables and because of the non-normal distribution of the spirituality scale. The models were adjusted for gender, age and socioeconomic status, because these variables often represent potential confounders in adolescent research. Each of the independent variables was tested in a separate model. From the whole sample, 402 (9.6%) participants reported that they do not have or see their father, and 86 (2.1%) reported that they do not have or see their mother. These respondents were excluded from the corresponding analyses. Finally, the analyses were also repeated for religious attendance and spirituality mutually adjusted (Model 3) and in interaction (Model 4). In order to reduce the probability of an increased Type II error in multiple testing, the significance level was set to alpha = 0.01. For the sensitivity analysis using the dichotomized spirituality, the prevalence of the easiness of communication with parents and of perceived emotional support were compared with the proportion test (*z*-test). All analyses were performed using the statistical software package IBM SPSS version 21 (New York, NY, USA).

## 3. Results

The background characteristics of the sample are presented in Table 1. Of the respondents, 296 (7.1%) reported attending church services once a week or more, 399 (9.5%) scored in the upper quartile of the spirituality scale and for the purpose of sensitivity analysis were considered to be spiritual. The average spirituality score in the whole sample was 22.0. (SD = 7.61), a mean MSPSS score was 5.9 ± 1.30 and a mean fractional rank (ridit) score of FAS was 0.5 ± 0.29.

A comparison of the groups of attending and non-attending respondents did not reveal any significant differences regarding age, gender, or perceived emotional support. However, the groups differed significantly in their communication with both parents as well as in 5 of 9 kinds of parental monitoring behaviours. Moreover, the groups of attending and non-attending respondents differed significantly (*p* < 0.001) from each other regarding the level of spirituality. For attending respondents, the mean SWBS score was 32.0 with SD = 8.16 (RWB = 18.0 ± 5.89; EWB = 14.0 ± 3.35), while for non-attending the mean SWBS score was 21.3 ± 6.99 (RWB = 8.2 ± 5.27; EWB = 13.1 ± 3.77).

A comparison of the groups of spiritual and non-spiritual respondents showed a significantly higher prevalence of high spirituality (i.e., the upper quartile of a score) among boys (*p* < 0.05) compared to girls, among 7th grade students compared to the 9th grade (*p* < 0.001), among respondents who reported a high perceived emotional support (*p* < 0.001), and among respondents who reported a higher parental monitoring (6 of 9 behaviours, *p* < 0.001).

The results of Pearson correlation coefficients are depicted in Table 2. Religious attendance was positively correlated with spirituality (*r* = 0.34, *p* < 0.001), however, it showed only very weak (not exceeding 0.07) and mostly non-significant correlations with the dependent variables of the study. Spirituality showed similarly low values (below 0.16), with the exception of a significant correlation with perceived family support (*r* = 0.33, *p* < 0.001). Communication with mother was positively correlated with communication with father (*r* = 0.36, *p* < 0.01) and perceived emotional support (*r* = 0.41, *p* < 0.01). Communication with father was positively correlated with perceived emotional support (*r* = 0.30, *p* < 0.01). However, correlations of these three variables with the nine types of parental monitoring behaviour were either non-significant or weak, i.e., r did not exceed 0.15.

About 81% of respondents reported the finding their communication with mother easy, the figure being lower for communication with father (about 63%). Approximately three out of four of the participants also reported high perceived emotional support from their families. Parental monitoring ranged for different kinds of behaviour from 26.9% to 95.7%, with the control for smoking being the highest.

Table 3 shows the results of the logistic regression adjusted for gender and age, aimed at the association between both religious attendance (Model 1) and spirituality (Model 2), with the ease of communication in the family and the perceived emotional support. Compared to non-attending respondents, attending respondents were less likely to report easy communication with mother (*p* < 0.01), while the other associations were not significant. In contrast, compared to non-spiritual respondents, spiritual respondents were more likely to report both easier communication with parents (*p* < 0.001) and a higher perceived emotional support (OR per SD change 1.73, 99% CI 1.55–1.92; *p* < 0.001). However, as further analysis showed, easier communication with parents was associated only with adolescents’ existential well-being (*p* < 0.001), while no significant results were found for their religious well-being. Both subscales were associated with higher perceived emotional support (*p* < 0.001).

**Figure 1:**
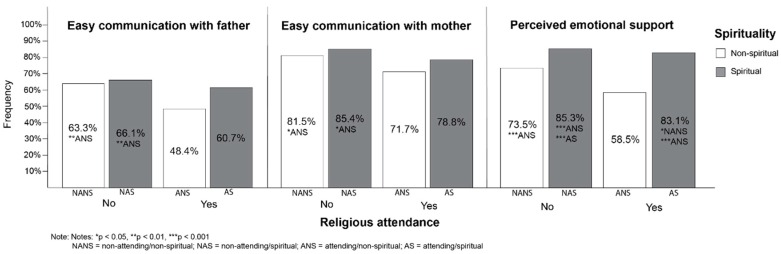
Prevalence of adolescent communication with parents and of perceived emotional support in groups with different combinations of religious attendance and spirituality.

Model 3 shows that after mutual adjustment, both religious attendance and spirituality were significantly associated with all three dependent variables (*p* < 0.001), i.e., communication with father, communication with mother, and perceived emotional support, with the figures decreasing for religious attendance and increasing for spirituality. Nevertheless, the interaction of both variables (Model 4) was not significant in any of the observed variables. Sensitivity analysis using dichotomized spirituality (see Figure 1) suggests that the subgroup which perceived difficulties in communication with parents and a lack of emotional support were the respondents who were attending, but not spiritual.

Table 4 depicts associations between adolescent parental monitoring of different kinds of behaviour with adolescent religious attendance and spirituality. The parents of attending respondents (Model 1) were more likely to control adolescents’ computer games playing, their time on the Internet) (*p* < 0.001), and their regular breakfast consumption (*p* < 0.001), whereas they were less likely to allow eating meals in front of the screen (*p* < 0.001) compared to the parents of non-attending respondents.

Spirituality (Model 2) showed a similar pattern, with a significant increase of the likelihood of parental monitoring in the case of screen-based activities (watching TV, playing computer games, spending time on the Internet) (*p* < 0.001), a regular breakfast consumption (*p* < 0.001), eating in front of the screen (*p* < 0.001), sweets and soft drinks consumption (*p* < 0.001). Regarding the associations with the subscales, the RWB was associated with five of the observed behaviour (*p* < 0.001), whereas the EWB with three of them (*p* < 0.05 to *p* < 0.001).

Mutual adjustment of religious attendance and spirituality (Model 3) revealed, in most cases, similar figures for both variables as previous analyses; however, in some cases it lost significance for religious attendance. The interaction of religious attendance and spirituality was not significant in any of the observed variables.

## 4. Discussion

We explored the association between some family characteristics (family communication, perceived emotional support, and parental monitoring style) and adolescent religious attendance and spirituality in a highly secular country. We found that religious attendance was significantly associated with more difficult communication with mother. In contrast, spiritual respondents were more likely to report both easier communication with parents and good perceived emotional support. Regarding parental monitoring, adolescents reported that the parents of attending respondents were in four of the nine observed behaviours more likely to be in control. The same held for spirituality, where the results were significant in five behaviours. The RWB was associated with higher parental monitoring in five behaviours, the EWB in three.

In our study, we did not find any significant differences in religious attendance between genders, and moreover, we found a higher prevalence of a high spirituality among boys. These findings are in contrast with the recent findings of studies on representative Czech samples which reported a higher prevalence of R/S among women [35,36] in adults. Moreover, yet another study on Czech adolescents came to an opposite conclusion [37]. However, it is also possible that these discrepancies reflect the use of different measurement tools. The other questionnaires may have used items with a stronger emotional content, and this content might be better accepted by women than by men [38]. However, further research on Czech adolescent samples would be needed to confirm this hypothesis.

Our results showed that attending respondents were less likely to perceive their communication with mother as easy, while no significant results were found for communication with father or perceived emotional support. However, a sensitivity analysis suggests that the group with more difficult family relationships could be represented by attending non-spiritual respondents. Previous research has usually reported a positive association between the religiosity of parents or adolescents and a higher family satisfaction in the relationship [39,40], which contradicts our findings. One explanation for this disparity is that family satisfaction might not totally correspond to the level and quality of family communication [41]. However, it is also important to note that most studies on adolescent R/S have been performed in predominantly religious countries. Therefore, it is also possible that the Czech secular environment may play its role. This might be true especially for adolescents. Within our age group, it is also possible that adolescent religious attendance is still a part of family tradition, and so attending non-spiritual respondents may go to church more to fulfil parental expectations than because of their own inner convictions. Especially in a secular environment, this situation might be demanding for adolescents who are surrounded by non-religious peers. Therefore, it seems that without a proper inner content, a higher emphasis on rules and maintaining traditions may hinder mutual openness and communication in the family. Some authors have also pointed out that when adolescents become more or less religious than their parents, their mutual communication could be influenced negatively [42]. This explanation could also be supported by the studies which reported a higher risk of estrangement of adult children who hold different values than their mothers [43] and studies reporting possible deleterious effects of family arguments about religion on child development [44]. Moreover, our previous research showed that the discrepancy in adolescent religious attendance and spirituality is associated with a higher occurrence of a health-risk behaviour [10], which could in turn further deteriorate family relationships in traditional religious families.

We also found that although spirituality was generally associated with easy communication with parents, it was only the association with the EWB subscale that was statistically significant. It suggests that the existential aspect of spirituality (a sense of hope and meaning in life) was in the Czech conditions more important in connection with family communication than the explicit relationship with God. Given the recent trend in research that links the attachment theory and the image of God [45,46], our results contradict the presumption that respondents who have a close and safe relationship with their parents will also report a positive relationship to God and vice versa. An explanation for these divergent findings could be that the RWB might more reflect the adolescents’ set of beliefs and rational knowledge about God, the so-called God concept [47], than their emotional experience of that relationship.

Our findings regarding parental monitoring are in line with the general findings in this area, in which higher parental supervision of religious adolescents is reported [40], and could also contribute to explaining the lower risk of excessive screen-based activities that have already been described for attending adolescents [31]. A possible explanation of this finding is that religious norms encourage parents to invest into their children in order to “train up a child” [44]. At the same time, the feeling of being controlled could negatively influence the adolescents’ communication with their parents, as found in our results.

Based on the family communication patterns theory [11], Czech religious families, especially those with lower emphasis on the spiritual dimension, possibly might correspond to “protective families”, with a high emphasis on obedience to authorities and a low level of family communication. In contrast, some of the families of Czech spiritual respondents possibly might correspond to the “consensual type”, with a high emphasis on conversation and conformity orientation, where children usually feel strong emotional support.

### 4.1. Strengths and Limitations

This study has several important strengths, the most important being the large and representative sample size of adolescents, the high response rate, and the use of the well-established HBSC methodology. Another strength is that this study extends the knowledge of the adolescents’ family conditions by assessing the role of religious attendance and spirituality. One limitation is that the design of the study did not allow us to measure the religious attendance or spirituality of the parents directly, and, also, the parental monitoring was reported by the adolescents only. Other limitations might be a potential information bias, as our data were based on self-reports of adolescents, which can be inaccurate or influenced by social desirability, and the cross-sectional design of the study, which does not allow us to make conclusions on causality. Moreover, it is also possible that problems in family communication might be influenced by other variables (e.g., quality of parental bonding), which were not assessed in this study.

### 4.2. Implications

Our research suggests that adolescent spirituality, especially some of its positive effects, like a perceived meaning of one’s life, might contribute to good adolescent communication with their parents and emotional support. Adolescents might therefore also be encouraged to develop this aspect of their lives. However, on the contrary, dissonance in this area, i.e., adolescent religious attendance without their own internal convictions, can possibly result in more difficult family communication. Religious parents should be better informed about these risks and the possible negative consequences of a mere maintaining of traditional values without their internal dimension.

Further research should therefore focus on understanding the causes and consequences of discrepancies in adolescent–parent religious and spiritual values. It could also examine the role of the parental religiosity/spirituality in order to complete the present results and should take into account other possible confounding variables.

## 5. Conclusions

We found that religious attendance was associated with lower self-reported easiness of communication with parents. In contrast, spirituality was associated with easier communication with parents and more perceived emotional support. Parents of attending as well as of spiritual respondents were in general more likely to control adolescent behaviour. Further research should assess the association of the dissonance of adolescent–parent religiosity/spirituality and higher parental monitoring with the lower easiness of family communication.

## Figures and Tables

**Table 1:** Sample characteristics by religious attendance and spirituality.

	Number	%	Missing Cases Per Variable Cases Per Variable	Religious Attendance				*p*-Value	Spirituality				*p*-Value
				Attending ^a^		Non-Attending			Spiritual ^b^		Non-Spiritual		
				Number	%	Number	%		Number	%	Number	%	
Total	4182	100		296	7.1	3886	92.9						
Gender								*n.s.*					*p* < 0.05
Boys	2034	48.6	0	131	44.3	1903	49.0		213	53.4	1821	48.1	
Girls	2148	51.4	0	165	55.7	1983	51.0		186	46.6	1962	51.9	
Age								*n.s.*					*p* < 0.001
13 years old (7th grade)	2091	50.0	0	146	49.3	1945	50.1		245	61.4	1846	48.8	
15 years old (9th grade)	2091	50.0	0	150	50.7	1941	49.9		154	38.6	1937	51.2	
Easy communication with parents ^c^													
With father	2342	62.8	452	148	55.0	2194	63.4	*p* < 0.01	232	63.9	2110	62.7	*n.s.*
With mother	3290	81.4	138	210	75.5	3080	81.8	*p* < 0.05	318	82.8	2972	81.2	*n.s.*
Perceived emotional support ^d^	3092	74.1	7	211	71.3	2881	74.3	*n.s.*	337	84.5	2755	73.0	*p* < 0.001
Parental monitoring ^e^													
Watching TV	1122	26.9	17	100	34.0	1022	26.4	*p* < 0.01	149	37.3	973	25.8	*p* < 0.001
Playing PC games	1533	36.9	34	143	49.3	1390	36.0	*p* < 0.001	194	49.1	1339	35.6	*p* < 0.001
Time on the Internet	1191	28.7	35	113	38.6	1078	27.9	*p* < 0.001	159	39.8	1032	27.5	*p* < 0.001
Being out after school	2087	50.3	35	156	53.1	1931	50.0	*n.s.*	211	53.0	1876	50.0	*n.s.*
Obligatory breakfast	1898	45.8	43	166	56.5	1732	45.0	*p* < 0.001	220	55.7	1678	44.8	*p* < 0.001
Eating in front of the screen	1846	44.5	40	162	55.5	1684	43.7	*p* < 0.001	217	54.8	1629	43.5	*p* < 0.001
Sweets and soft drink consumption	2074	49.9	31	159	54.3	1915	49.6	*n.s.*	211	53.1	1863	49.6	*n.s.*
Smoking (9th grade only)	1986	95.7	20	143	96.0	1843	95.7	*n.s.*	145	94.8	1841	95.8	*n.s.*
Alcohol consumption (9th grade only)	1870	90.0	15	141	94.6	1729	89.6	*n.s.*	136	89.5	1734	90.0	*n.s.*

Notes: ^a^ Attending at least once a week, ^b^ SWBS score 34 or higher. ^c^ Only numbers regarding the respondents who reported good communication with parents are presented. ^d^ Only numbers regarding the respondents who reported high perceived family support (mean MSPSS ≥ 5.5) are presented. ^e^ Only numbers regarding the respondents whose parents were monitoring for each kind of behaviour are presented.

**Table 2:** Correlations of the variables in the study.

	Spirituality ^a^	Relig. Attendance	Age ^a^	Gender	Communication		Perceived Emotional Support ^a^	Parental Monitoring							
					With Father	With Mother		Watching TV	Playing PC Games	Time on the Internet	Being Out after School	Obligatory Breakfast	Eating in Front of the Screen	Sweets and Soft Drinks Cons.	Smoking ^b^
Religious attendance	0.34 ***														
Age ^a^	−0.08 ***	0.00													
Gender	−0.06 ***	0.02	−0.05 **												
Easy communication															
With father	0.10 ***	−0.05 **	−0.04 *	−0.19 ***											
With mother	0.16 ***	−0.04 **	−0.03	−0.06 ***	0.36 ***										
Perceived emotional support ^a^	0.33 ***	−0.03	−0.06 ***	-0.10 ***	0.30 ***	0.41 ***									
Parental monitoring															
Watching TV	0.06 ***	0.06 **	−0.17 ***	−0.02	0.00	−0.01	0.04 *								
Playing PC games	0.08 ***	0.07 **	−0.22 ***	−0.14 ***	0.04 *	0.01	0.04 *	0.64 ***							
Time on the Internet	0.06 ***	0.04 **	−0.20 ***	−0.06 ***	0.05 **	0.01	0.05 **	0.51 ***	0.56 ***						
Being out after school	0.01	0.02	−0.13 ***	0.14 ***	−0.03	−0.02	−0.03	0.24 ***	0.20 ***	0.22 ***					
Obligatory breakfast	0.12 ***	0.06 ***	−0.11 ***	−0.05 ***	0.10 **	0.08 ***	0.16 ***	0.21 ***	0.21 ***	0.18 ***	0.10 ***				
Eating in front of the screen	0.09 ***	0.06 ***	−0.11 ***	−0.00	0.03	0.02	0.03 *	0.16 ***	0.16 ***	0.15 ***	0.04 **	0.12 ***			
Sweets and soft drink consumption	0.08 ***	0.02	−0.17 ***	0.04 **	0.02	0.04 *	0.05 **	0.16 ***	0.18 ***	0.17 ***	0.06 ***	0.14 ***	0.31 ***		
Smoking ^b^	0.05 *	0.00	−0.08 ***	0.02	−0.00	0.03	0.03	−0.00	0.02	−0.03	−0.02	0.06 *	0.04 *	0.08 ***	
Alcohol consumption ^b^	0.01	0.04	−0.05 *	−0.02	−0.01	0.00	0.01	0.03	0.07 **	0.02	0.04 *	0.06 **	0.14 ***	0.11 ***	0.30 ***

Notes: * *p* < 0.05, ** *p* < 0.01, *** *p* < 0.001. ^a^ Assessed as a scale variable ^b^ 9th grade only.

**Table 3:** Associations of adolescent perceive easiness of communication with parents and perceived emotional support with religious attendance and spirituality (standardized to *z*-scores), adjusted for age, gender, and socioeconomic status (odds ratios, OR, and 99% confidence intervals, CI).

	Communication with Father	Communication with Mother	Perceived Emotional Support
	OR (99% CI)	OR (99% CI)	OR (99% CI)
Model 1: Religious attendance			
Non-attending	1 (ref)	1 (ref)	1 (ref)
Attending	0.72 (0.51–1.003) *	0.68 (0.47–0.99) **	0.87 (0.61–1.23)
Model MSPSS.2: Spirituality (per SD) ^a^			
SWBS ^b^	1.12 (1.02–1.23) **	1.38 (1.23–1.55) ***	1.73 (1.55–1.92) ***
RWB ^c^	0.97 (0.89–1.06)	1.05 (0.95–1.17)	1.17 (1.06–1.29) ***
EWB ^d^	1.33 (1.21–1.45) ***	1.65 (1.49–1.83) ***	2.10 (1.90–2.31) ***
Model 3: Religious attendance and spirituality mutually adjusted			
Attending vs. non-attending	0.55 (0.38–0.80) ***	0.36 (0.24–0.55) ***	0.35 (0.23–0.52) ***
Spirituality (per SD)	1.19 (1.08–1.32) ***	1.54 (1.36–1.74) ***	1.90 (1.70–2.14) ***
Model 4: Interaction of religious attendance and spirituality			
Attendance vs. non-attendance	0.59 (0.35–1.02) *	0.49 (0.28–0.87) **	0.32 (0.19–0.54) ***
Spirituality (per SD)	1.20 (1.08–1.33) ***	1.60 (1.40–1.83) ***	1.88 (1.66–2.13) ***
Attendance x spirituality	0.95 (0.68–1.32)	0.74 (0.51–1.07)	1.11 (0.77–1.59)

Notes: * *p* < 0.05, ** *p* < 0.01, *** *p* < 0.001. Missing cases per communication with father: *n* = 503, communication with mother: *n* = 190, perceived emotional support: *n* = 64. ^a^ standardized to z scores; SD = standard deviation; ^b^ SWBS = Spiritual Well-being Scale; ^c^ RWB = Religious Well-Being Subscale; ^d^ EWB = Existential Well-Being Subscale.

**Table 4:** Associations of adolescent parental monitoring (control of behaviour) with religious attendance and spirituality (standardized to z-scores), adjusted for age, gender and socioeconomic status (odds ratios and 99% confidence intervals).

	Watching TV	PC Games Playing	Time on the Internet	Being out after School	Obligatory Breakfast
Model 1: Religious attendance					
Non-attending	1 (ref)	1 (ref)	1 (ref)	1 (ref)	1 (ref)
Attending	1.42 (1.00–2.00) *	1.81 (1.29–2.52) ***	1.60 (1.15–2.24) ***	1.10 (0.79–1.52)	1.57 (1.14–2.16) ***
Model 2: Spirituality (per SD) ^a^					
SWBS ^b^	1.13 (1.03–1.24) **	1.17 (1.07–1.28) ***	1.18 (1.08–1.30) ***	1.05 (0.96–1.14)	1.23 (1.13–1.33) ***
RWB ^c^	1.16 (1.06–1.27) ***	1.18 (1.08–1.29) ***	1.22 (1.11–1.33) ***	1.06 (0.97–1.15)	1.13 (1.05–1.23) ***
EWB ^d^	1.01 (0.92–1.11)	1.05 (0.96–1.15)	1.03 (0.94–1.13)	1.01 (0.93–1.09)	1.24 (1.14–1.35) ***
Model 3: Religious attendance and spirituality mutually adjusted					
Attending vs. non-attending	1.22 (0.84–1.78)	1.54 (1.07–2.20) **	1.31 (0.91–1.89)	1.03 (0.73–1.46)	1.21 (0.85–1.71)
Spirituality (per SD)	1.11 (1.01–1.23) **	1.12 (1.02–1.23) **	1.15 (1.05–1.27) ***	1.04 (0.95–1.14)	1.21 (1.10–1.32) ***
Model 4: Interaction of religious attendance and spirituality					
Attendance vs. non-attendance	1.05 (0.58–1.90)	1.43 (0.84–2.41)	1.14 (0.65–2.00)	0.92 (0.56–1.53)	1.05 (0.63–1.75)
Spirituality (per SD)	1.10 (0.99–1.22) *	1.12 (1.01–1.23) **	1.14 (1.03–1.26) **	1.03 (0.94–1.14)	1.19 (1.09–1.31) ***
Attendance x spirituality	1.12 (0.79–1.59)	1.07 (0.77–1.47)	1.12 (0.80–1.57)	1.10 (0.81–1.50)	1.13 (0.82–1.54)
	**Eating in Front of the Screen**	**Sweets and Soft Drinks Consumption**	**Smoking (15 Years Old)**	**Alcohol Use (15 Years Old)**	
Model 1: Religious attendance					
Non-attending	1 (ref)	1 (ref)	1 (ref)	1 (ref)	
Attending	1.60 (1.16–2.20) ***	1.25 (0.90–1.72)	1.10 (0.36–3.35)	2.09 (0.80–5.45) *	
Model 2: Spirituality (per SD) ^a^					
SWBS	1.18 (1.09–1.28) ***	1.13 (1.04–1.22) ***	1.16 (0.86–1.57)	1.05 (0.86–1.29)	
RWB	1.17 (1.07–1.27) ***	1.07 (0.99–1.16) *	1.04 (0.78–1.40)	1.02 (0.83–1.24)	
EWB	1.10 (1.01–1.19) **	1.15 (1.05–1.24) ***	1.24 (0.94–1.63) *	1.08 (0.89–1.31)	
Model 3: Religious attendance and spirituality mutually adjusted					
Attending vs. non-attending	1.31 (0.93–1.85) *	1.06 (0.75–1.50)	0.88 (0.27–2.92)	2.13 (0.78–5.83)	
Spirituality (per SD)	1.15 (1.05–1.26) ***	1.12 (1.02–1.22) **	1.18 (0.85–1.63)	0.99 (0.79–1.23)	
Model 4: Interaction of religious attendance and spirituality					
Attendance vs. non-attendance	1.31 (0.79-2.15)	0.99 (0.60–1.64)	0.57 (0.17–1.86)	1.82 (0.46–7.27)	
Spirituality (per SD)	1.15 (1.05–1.26) ***	1.11 (1.02–1.22) **	1.07 (0.76–1.50)	0.98 (0.78–1.23)	
Attendance x spirituality	1.003 (0.74–1.36)	1.06 (0.79–1.44)	2.28 (0.83–6.23) *	1.15 (0.46–2.88)	

Notes: * *p* < 0.05, ** *p* < 0.01, *** *p* < 0.001; Missing cases per analysis: Watching TV: *n* = 73, PC games playing: *n* = 88, Time on the internet: *n* = 86, Being out after school: *n* = 86, Obligatory breakfast: *n* = 96, Eating in front of the screen: *n* = 94, Sweets and soft drinks consumption: *n* = 83, Smoking (15 years old): *n* = 2118, Alcohol use (15 years old): *n* = 2115. ^a^ standardized to *z* scores; ^b^ SWBS = Spiritual Well-being Scale; ^c^ RWB = Religious Well-Being Subscale; ^d^ EWB = Existential Well-Being Subscale.
